# Grk7 but not Grk1 undergoes cAMP-dependent phosphorylation in zebrafish cone photoreceptors and mediates cone photoresponse recovery to elevated cAMP

**DOI:** 10.1016/j.jbc.2022.102636

**Published:** 2022-10-21

**Authors:** Jared D. Chrispell, Yubin Xiong, Ellen R. Weiss

**Affiliations:** Department of Cell Biology and Physiology, The University of North Carolina at Chapel Hill, Chapel Hill, North Carolina, USA

**Keywords:** Cyclic AMP, Retina, Photoreceptors, Phototransduction, Protein kinase A, Protein phosphorylation, G protein-coupled receptor, G protein-coupled receptor kinase 1, G protein-coupled receptor kinase 7, Zebrafish, Electroretinogram, Forskolin, APB, 2-amino-4-phosphonobutyric acid, DMSO, dimethyl sulfoxide, ERG, electroretinogram, HRMA, high resolution melt analysis, ISI, interstimulus interval, sgRNA, single-guide RNA

## Abstract

In the vertebrate retina, phosphorylation of photoactivated visual pigments in rods and cones by G protein–coupled receptor kinases (GRKs) is essential for sustained visual function. Previous *in vitro* analysis demonstrated that GRK1 and GRK7 are phosphorylated by PKA, resulting in a reduced capacity to phosphorylate rhodopsin. *In vivo* observations revealed that GRK phosphorylation occurs in the dark and is cAMP dependent. In many vertebrates, including humans and zebrafish, GRK1 is expressed in both rods and cones while GRK7 is expressed only in cones. However, mice express only GRK1 in both rods and cones and lack GRK7. We recently generated a mutation in *Grk1* that deletes the phosphorylation site, Ser21. This mutant demonstrated delayed dark adaptation in mouse rods but not in cones *in vivo*, suggesting GRK1 may serve a different role depending upon the photoreceptor cell type in which it is expressed. Here, zebrafish were selected to evaluate the role of cAMP-dependent GRK phosphorylation in cone photoreceptor recovery. Electroretinogram analyses of larvae treated with forskolin show that elevated intracellular cAMP significantly decreases recovery of the cone photoresponse, which is mediated by Grk7a rather than Grk1b. Using a cone-specific dominant negative PKA transgene, we show for the first time that PKA is required for Grk7a phosphorylation *in vivo*. Lastly, immunoblot analyses of rod *grk1a−/−* and cone *grk1b−/−* zebrafish and *Nrl−/−* mouse show that cone-expressed Grk1 does not undergo cAMP-dependent phosphorylation *in vivo*. These results provide a better understanding of the function of Grk phosphorylation relative to cone adaptation and recovery.

In vertebrates, visual interpretation of the surrounding environment relies on two types of specialized neurons in the retina: rod and cone photoreceptors. Rods are highly sensitive, requiring capture of only a single photon to initiate hyperpolarization of the photoreceptor (1, 2). However, rods are easily saturated and desensitized under conditions of prolonged brightness, thus providing vision mostly under low light conditions. Cones, while less sensitive than rods, recover more rapidly and are less prone to desensitization, which allows them to operate under a broader range of light intensities to provide higher visual acuity under bright light (3, 4). Despite these differences between rods and cones, the mechanisms of phototransduction in these cells are analogous: the retinoid chromophore bound to rhodopsin or cone opsin undergoes isomerization upon absorption of a photon, which induces a conformational change in the opsin allowing it to activate a G-protein (rod or cone transducin), which in turn activates a phosphodiesterase (PDE6) leading to a decrease in intracellular cGMP levels and closure of cGMP-gated cation channels in the cell membrane (5, 6). Sustained vision requires that the activated opsin return to the dark-adapted state to be sensitive to new stimuli. The first step in this regeneration process is phosphorylation of the opsin, which allows the binding of arrestin and results in steric hindrance of G protein binding (7). This phosphorylation is accomplished by the retina-specific members of the G protein–coupled receptor kinase (GRK) family, GRK1 and GRK7 (8, 9, 10, 11, 12).

The retinal GRKs possess several qualities that govern their capacity to modulate the recovery of visual pigments to their ground state. In addition to an interaction of GRK1 with the neuronal calcium sensor protein recoverin (13, 14, 15), human recombinant GRK1 and GRK7 are phosphorylated by cAMP-dependent PKA *in vitro* at Ser21 and Ser36, respectively (16). Observations in several vertebrate models reveal that both GRK1 and GRK7 display elevated levels of phosphorylation at these sites *in vivo* under dark-adapted conditions when intracellular levels of cAMP are elevated in photoreceptors (17, 18, 19, 20, 21, 22). *In vivo* phosphorylation of GRK1 was independent of phototransduction based on the observation that phosphorylation was low in the light and high in the dark in *gnat1−/−* mice (18). Additionally, *in vitro* analysis demonstrates that GRK1 and GRK7 phosphorylated at these residues have decreased kinase activity that impairs their ability to phosphorylate rhodopsin (16).

Expression of the retinal GRKs is heterogeneous in vertebrates but in many, such as humans, GRK1 is expressed in both rods and cones while GRK7 is expressed only in cones (23, 24). Some species, like pigs and dogs, only express GRK1 in rods and only GRK7 in cones, whereas rats and mice have lost the gene for GRK7 and express only GRK1 in both rods and cones (25). In humans, inactivating mutations in GRK1 result in Oguchi disease, a form of stationary night blindness characterized by slow dark adaptation (26, 27). Patients with Enhanced S-Cone Syndrome possess L/M-cones which express only GRK1 and display a mild delay in recovery, but have S-cones that lack both GRKs and exhibit extremely delayed recovery (28). We previously reported that in zebrafish, which possess a retinal GRK expression pattern similar to humans, both Grk1b and Grk7a contribute to recovery of the cone photoresponse, confirming the participation of both kinases in the return of cone photoreceptors to the dark-adapted state (29). We also observed that dark adaptation is significantly decreased *in vivo* in the rods of mice expressing GRK1 with the mutation Ser21Ala, which blocks phosphorylation (30). This suggests a role for cAMP-dependent phosphorylation of GRK1 in modulating the lifetime of activated rhodopsin. Dark adaptation was unaffected in the cones of the GRK1-S21A mice, which is surprising considering that mice express the same GRK1 protein in both rod and cone photoreceptors.

In this report, we evaluate the effects of elevated cAMP on phosphorylation of GRK1 and GRK7 in cones as well as recovery of the cone photoresponse. We incubated WT and Grk-KO zebrafish larvae with forskolin (FSK), a potent activator of adenylyl cyclase that has been observed to increase intracellular cAMP in vertebrate photoreceptors (31, 32), and found that increased intracellular cAMP is associated with a significant delay in cone recovery only when Grk7a is expressed in cones. We also used a cone-specific dominant negative PKA transgenic zebrafish to show that PKA is part of the endogenous kinase pathway responsible for Grk7a phosphorylation in response to elevated cAMP. Finally, we analyzed the rod/cone GRK1 phosphorylation profile of the vertebrate retina using a newly created *grk1a−/−* zebrafish (which lacks the rod-expressed zebrafish Grk1a paralog), as well as the *Nrl−/−* mouse (which possesses an ‘all-cone’ retina), and found that GRK1 natively expressed in cones fails to undergo cAMP-dependent phosphorylation in both vertebrate species unlike GRK1 expressed in rods.

## Results

### Zebrafish Grk1 is phosphorylated *in vitro* by PKA and in dark-adapted zebrafish

**Figure 1:**
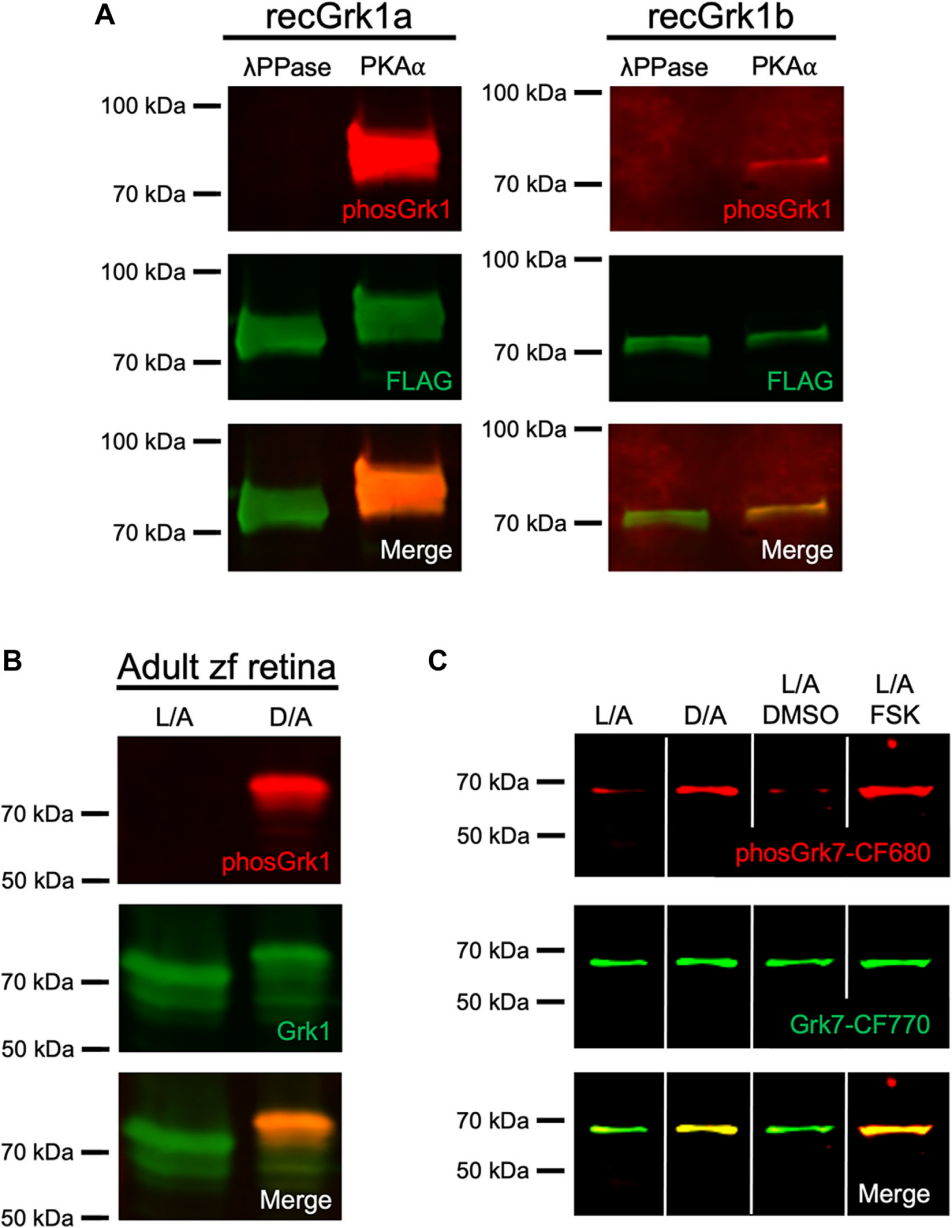
**Immunospecificity of antibodies against phosphorylated Grk1 and Grk7.***A*, FLAG-tagged, purified recombinant zebrafish Grk1a and Grk1b treated *in vitro* with either λ phosphatase or PKA⍺ catalytic subunit were subjected to immunoblot analysis. Immunoblots were probed with antibodies against the FLAG-tag (*green*) and phosphorylated Grk1 (*red*) followed by incubation with secondary antibodies. *B*, Adult zebrafish were light- or dark-adapted, euthanized, and retinal homogenates subjected to immunoblot analysis. Immunoblots were probed with antibodies against Grk1 (*green*) and phosphorylated Grk1 (*red*), followed by incubation with secondary antibodies. *C*, Larvae at 5 dpf were light- or dark- adapted, followed by incubation with forskolin or vehicle (dimethyl sulfoxide [DMSO]) for 30 min. Larvae were euthanized and intact eyes were harvested for immunoblot analysis. Immunoblots were probed against Grk7 (*green*) and phosphorylated Grk7 (*red*) with anti-Grk7 antibody directly conjugated to CF770 and anti-phosphorylated Grk7 antibody directly conjugated to CF680. Panel *C* was spliced for clarity due to noncontiguous loading on the same gel.

**Supplemental Figure S1:**
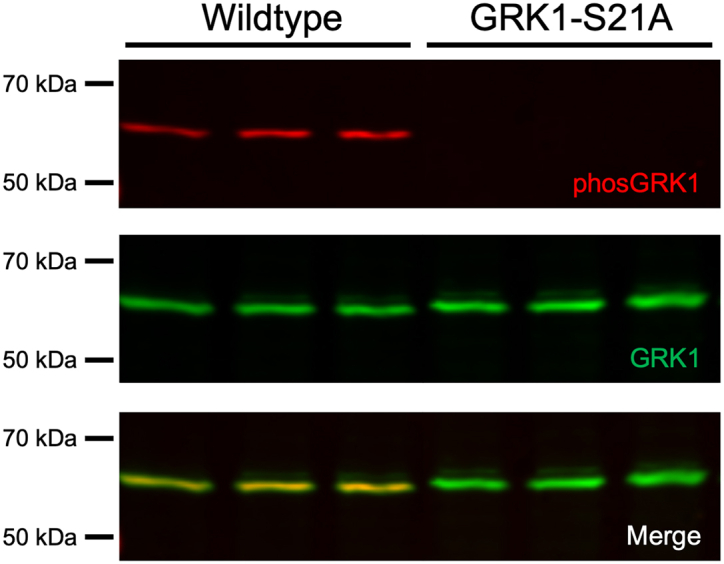
Immunospecificity of the anti-phosphorylated Grk1 antibody (generated against the zebrafish phosphopeptide) towards phosphorylated GRK1 in mice. Adult wildtype and GRK1-S21A mice were dark-adapted overnight, euthanized, and retinal homogenates subjected to immunoblot analysis using the same novel zebrafish-specific antibody against phosphorylated zebrafish Grk1. Immunoblots were probed with antibodies against total Grk1 (green) and phosphorylated Grk1 (red) followed by incubation with secondary antibodies.

Human recombinant GRK1 and GRK7 undergo phosphorylation by PKA⍺ *in vitro* at Ser21 and Ser36, respectively (16). Mouse GRK1 phosphorylation under dark-adapted conditions in a cAMP-dependent manner *in vivo* is detected using an antibody specific for GRK1 phosphorylated at Ser21 (18). However, the antibody used in these studies was generated against mouse sequence and performed poorly against phosphorylated zebrafish Grk1a and Grk1b (data not shown). To determine if Grk1 is phosphorylated in zebrafish, an antibody was generated against a peptide sequence corresponding to phospho-Ser21 in zebrafish Grk1, which is conserved in the Grk1a and Grk1b paralogs. To examine the immunospecificity of this antibody, recombinant FLAG-tagged Grk1a and Grk1b were expressed in HEK-293 cells and immunopurified with anti-FLAG beads. Purified FLAG-Grk1a and FLAG-Grk1b were then incubated *in vitro* with either λ phosphatase or PKA⍺ catalytic subunit and subjected to immunoblot analysis using the novel anti-phosGrk1 antibody and the anti-FLAG antibody. The anti-phosGrk1 antibody recognizes both recombinant Grk1a and Grk1b phosphorylated by the PKA⍺ catalytic subunit *in vitro* (Fig. 1*A*). This antibody also recognizes phosphorylated Grk1 in retinal homogenates from dark-adapted adult zebrafish (Fig. 1*B*). Additionally, the anti-phosGrk1 antibody generated against zebrafish peptides was capable of detecting endogenous phosphorylated GRK1 in dark-adapted WT mouse retina but not in GRK1-S21A mice, in which the cAMP-dependent phosphorylation site is mutated from a serine to an alanine (Fig. S1). These results demonstrate that the anti-phosGrk1 antibody recognizes cAMP-dependent Grk1 phosphorylation in both zebrafish and mice and does not discriminate between the zebrafish Grk1 paralogs.

The antibody against Grk7 was reported to recognize both paralogs of Grk7 in zebrafish, while the antibody against phosphorylated Grk7 showed cAMP-dependent phosphorylation of GRK7 at Ser36 (Ser33 in zebrafish) *in vivo* in multiple vertebrates *via* immunohistochemical analysis (17, 29). Since both antibodies are rabbit-derived, they were directly conjugated to distinct fluorophores. Immunoblot analysis confirms colocalization of bands corresponding to phosphorylated and total Grk7 in retinas of both dark-adapted and forskolin-treated larvae at 5 dpf (Fig. 1*C*).

### Both Grk1 and Grk7 are phosphorylated in retinas of zebrafish larvae incubated with forskolin

**Figure 2:**
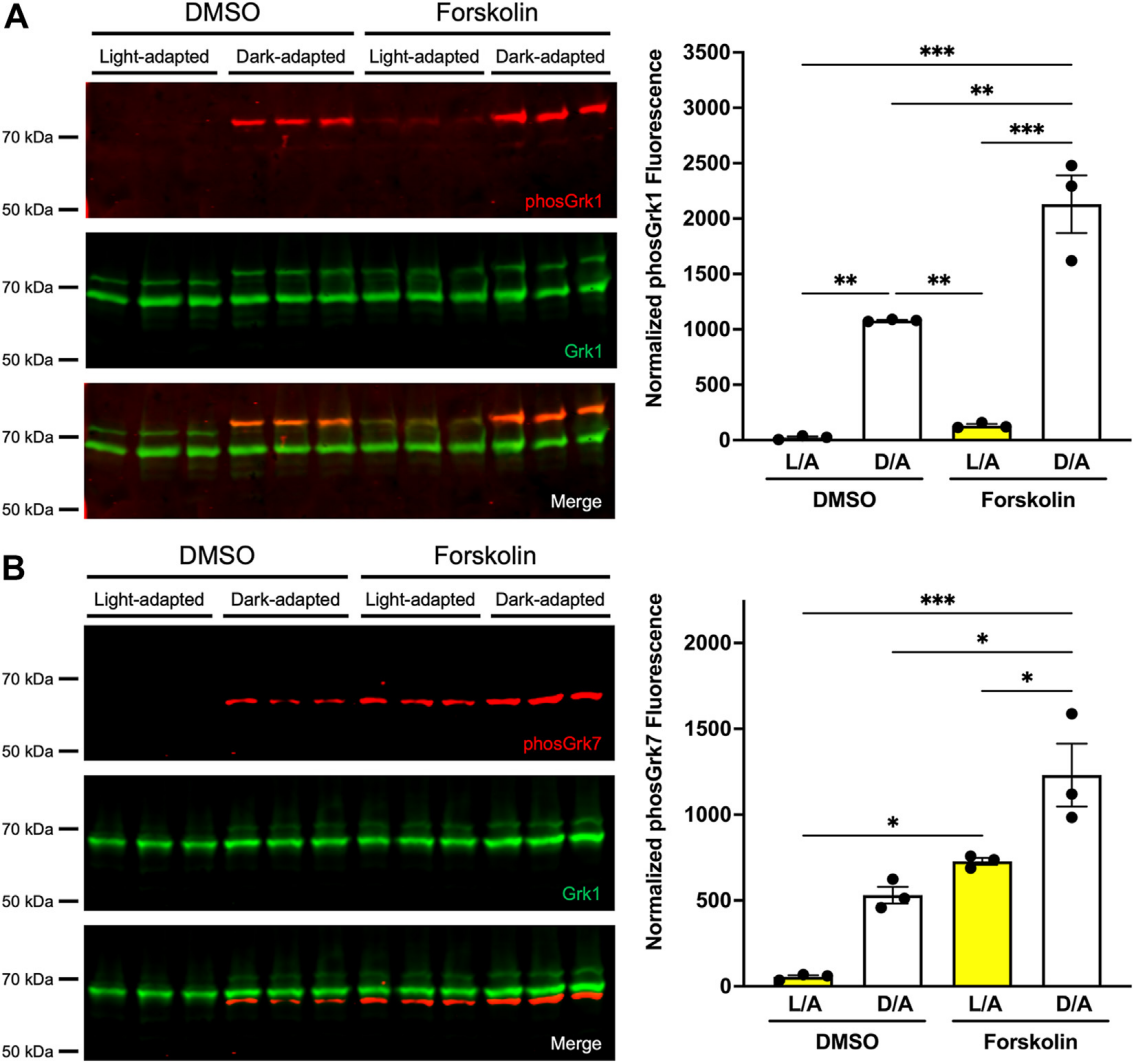
**Effect of light exposure and forskolin incubation on phosphorylation of retinal Grks in WT zebrafish larvae at 5 dpf.** Larvae were light- or dark-adapted, followed by incubation with forskolin or vehicle (DMSO, dimethyl sulfoxide) for 30 min. Larvae were euthanized and intact eyes were harvested for immunoblot analysis. Immunoblots were probed with antibodies against total Grk1 (*green*) and either (*A*) phosphorylated Grk1 (*red*) or (*B*) phosphorylated Grk7 (*red*) followed by incubation with secondary antibodies. The levels of phosphorylated Grk1 or Grk7 were normalized against total Grk1 and quantified. Statistical comparison of multiple groups was performed using a two-way ANOVA, followed by a Tukey post hoc test. Error bars represent SEM (n = 3). *p* ≤ 0.05 (∗), *p* ≤ 0.01 (∗∗), *p* ≤ 0.001 (∗∗∗).

Recombinant human GRK1 and GRK7 undergo phosphorylation in HEK-293 cells treated with forskolin (16). Similarly, frog retinas incubated in forskolin *ex vivo* display an increase in Grk7 phosphorylation (17). To determine the *in vivo* effects of increased cAMP on Grk phosphorylation in the zebrafish retina, larval zebrafish were incubated in forskolin under both light- and dark-adapted conditions prior to immunoblot analysis. Quantification of immunoblots show a significant increase in Grk1 phosphorylation under dark-adapted conditions compared to light-adapted conditions regardless of drug treatment and in dark-adapted larvae treated with forskolin compared to vehicle (DMSO, dimethyl sulfoxide) (Fig. 2*A*). We observed a 6-fold increase in Grk1 phosphorylation in light-adapted larvae treated with forskolin compared to those treated with vehicle as well as a phosphorylation-dependent electrophoretic mobility shift in Grk1 immunostaining in these larvae under these conditions. Immunoblot analysis and quantification also show a significant increase in Grk7 phosphorylation in larvae exposed to forskolin compared to vehicle regardless of background illumination (Fig. 2*B*). A significant increase in Grk7 phosphorylation was observed in forskolin-treated larvae under dark-adapted conditions compared to light adapted, and Grk7 phosphorylation was elevated 10-fold in vehicle-treated larvae under dark-adapted conditions compared to light adapted. These data suggest that under conditions where intracellular cAMP is elevated *in vivo*, both retinal Grks undergo phosphorylation.

### Recovery of the cone photoresponse is significantly delayed in zebrafish larvae exposed to forskolin

**Figure 3:**
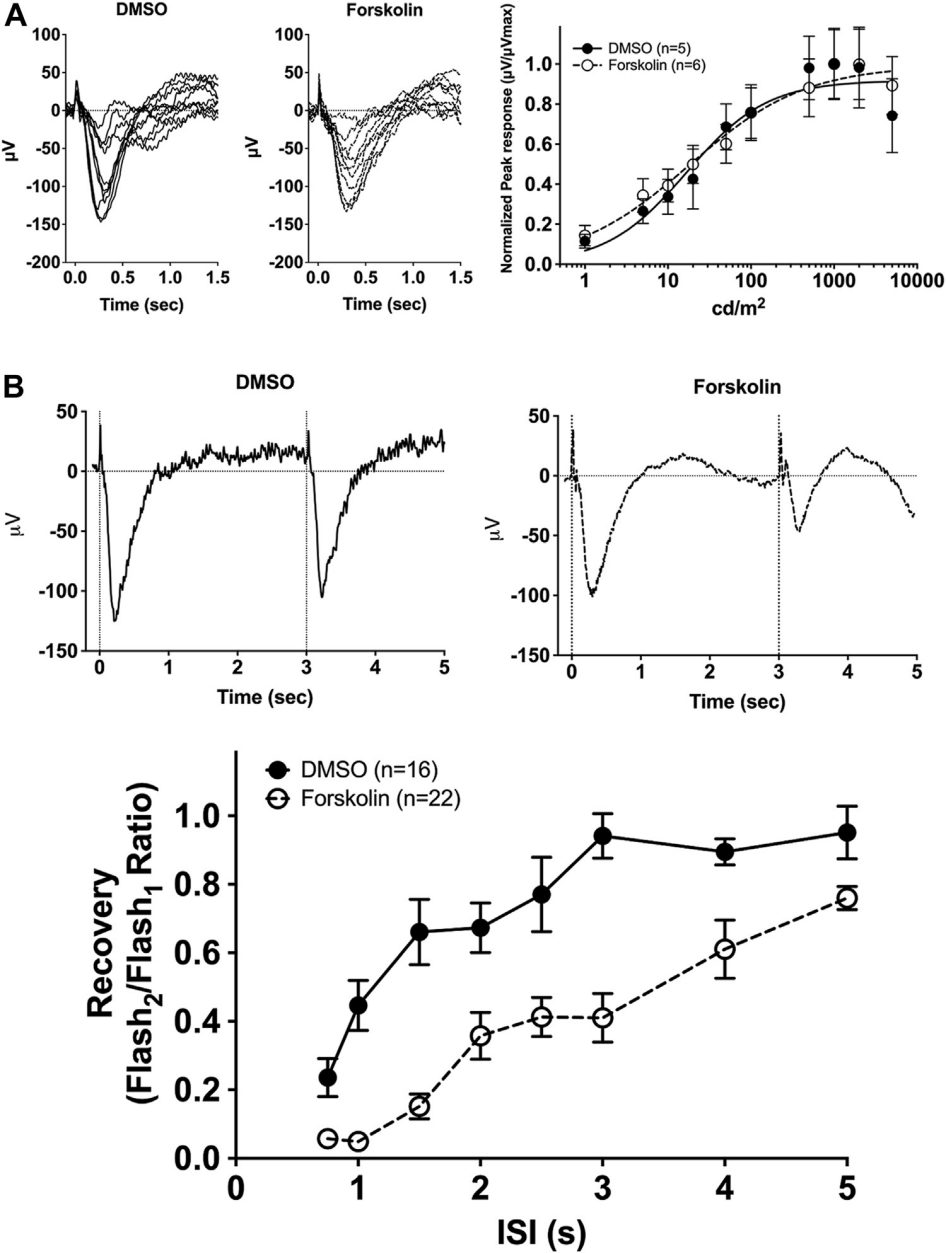
**Electrophysiological light responses, normalized sensitivity, and recovery of the cone-mass receptor potential in forskolin-treated WT larvae at 5 dpf.***A*, Representative electroretinogram (ERG) traces of isolated cone-mass receptor potentials in larvae incubated in vehicle (DMSO, dimethyl sulfoxide) or forskolin for 25 min, followed by a 5-min coincubation with 2-amino-4-phosphonobutyric acid (APB). Reponses were recorded under dark-adapted conditions to 20-ms flashes of light of increasing intensities from 0.1 cd/m^2^ to 5000 cd/m^2^. The fast initial positive deflection is attributed to a photovoltaic effect with the recording microelectrode. Mean-normalized peak response amplitudes were fit using the Naka–Rushton function. *B*, Representative ERG waveforms of treated larvae subjected to successive stimuli using a dual flash paradigm of a 20-ms flash of saturating white light (1000 cd/m^2^) with an interstimulus interval (ISI) of 3 s. *Vertical black dotted lines* indicate time of stimulus. Cone-mass receptor potential recovery was plotted as the ratio of the maximum isolated cone mass receptor potential response of the second stimulus to that of the initial stimulus for ISIs ranging from 0.75 s to 5 s. A linear mixed model analysis of covariance found a significant effect of forskolin compared to vehicle [F_(1, 350)_ = 115.6; *p* < 0.0001]. Error bars represent SEM.

To understand the effects of increased intracellular cAMP on the cone photoresponse, electroretinogram (ERG) recordings were performed on larvae incubated with forskolin. In larval zebrafish at 5 dpf, cones are electrophysiologically functional while nascent rods are not. The native photopic response at 5 dpf consists of a small a-wave elicited by the cone photoreceptors that is occluded by the larger b-wave response of the inner retina. Isolation of the cone mass receptor potential for recording was achieved through coincubation of larvae with 2-amino-4-phosphonobutyric acid (APB), an mGluR6 agonist, that blocks neurotransmission to ON bipolar cells and eliminates the b-wave (33, 34). Responses were recorded in sedated larvae across an intact eye to single 20-ms flashes of white light of intensities from 0.1 to 5000 cd/m^2^ (Fig. 3*A*, left panels). To compare cone sensitivity in WT larvae treated with either vehicle or forskolin, responses were normalized (μV/μV_Max_) and fit with the Naka–Rushton equation (Fig. 3*A*, right panel). No significant differences in peak amplitudes or normalized sensitivity were observed between vehicle- or forskolin-treated WT larvae.

To determine if exposure to forskolin affects the cone photoresponse recovery, ERG responses to successive stimuli with variable interstimulus intervals (ISI) were analyzed. Vehicle- or forskolin-treated WT larvae were subjected to a conditioning flash of saturating white light (1000 cd/m^2^, 20 ms), followed by a probe flash of the same intensity at varying ISIs. Recovery was measured as the ratio of the cone mass receptor potential of the probe flash to the initial conditioning flash. Representative recovery waveforms using an ISI of 3 s are shown in Figure 3*B* (top panels). A time course of the cone mass receptor potential recovery shows a statistically significant delay in recovery for forskolin-treated larvae compared to vehicle-treated larvae (F_(1, 350)_ = 115.6; *p* < 0.0001) (Fig. 3*B*, bottom panel). This indicates that increasing levels of intracellular cAMP via incubation of larvae with forskolin leads to impaired recovery of the cone photoresponse.

### Zebrafish larvae that lack Grk7a are insensitive to the forskolin-induced delay in recovery of the cone photoresponse

**Figure 4:**
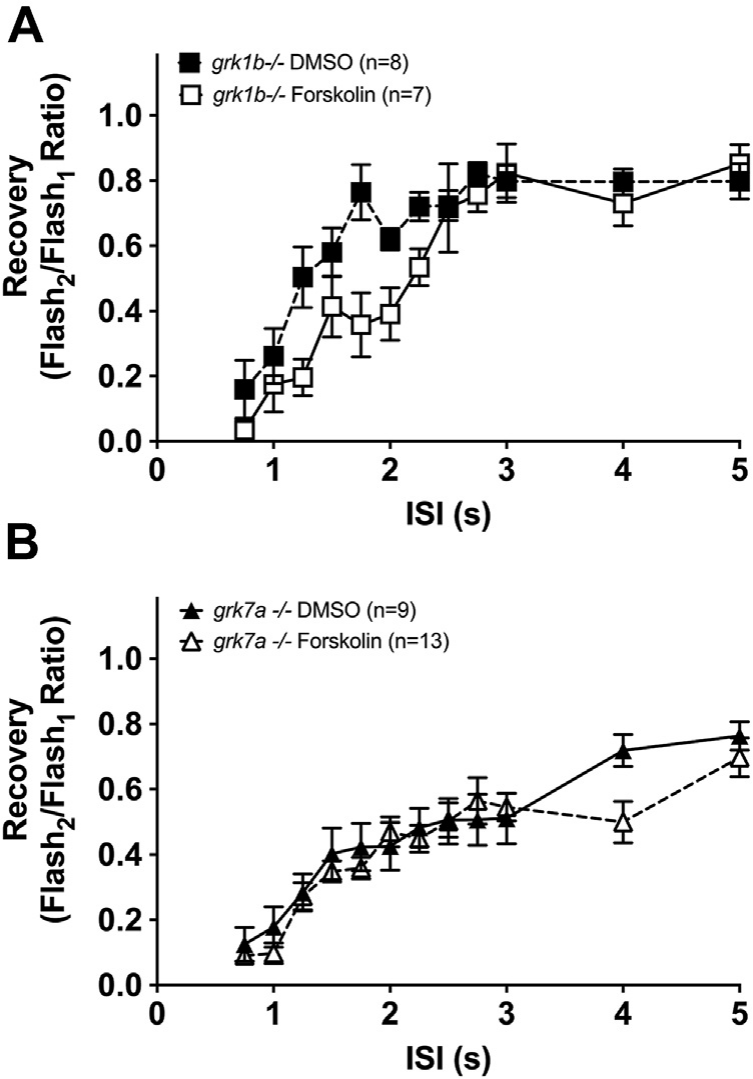
**Recovery of the cone-mass receptor potential in *grk* KO zebrafish larvae treated with forskolin.** Treated larvae were subjected to successive stimuli using a dual flash paradigm of a 20-ms flash of saturating white light (1000 cd/m^2^) with an interstimulus interval (ISI) ranging from 0.75 s to 5 s. Cone-mass receptor potential recovery was plotted as the ratio of the maximum isolated cone mass receptor potential response of the second stimulus to that of the initial stimulus for (*A*) *grk1b−/−* and (*B*) *grk7a−/−* larvae. A linear mixed model analysis of covariance found a significant effect of forskolin compared to vehicle (DMSO, dimethyl sulfoxide) for *grk1b−/−* (F_(1, 224)_ = 17.82; *p* <0.0001) larvae, but not *grk7a−/−* larvae (F_(1, 340)_ = 2.363; *p* = 0.1252). Error bars represent SEM.

We previously reported that both Grk1b and Grk7a contribute to the recovery of the cone photoresponse based on observations made using zebrafish lines in which either of the cone-specific Grk paralogs, Grk1b or Grk7a, was deleted through the use of transcription activator-like effector nucleases (29). To determine if one or both GRK paralogs mediate the delayed cone recovery observed when intracellular cAMP levels are elevated, paired flash ERG analysis was performed on *grk1b−/−* and *grk7a−/−* larvae treated with forskolin. In *grk1b−/−* larvae treated with forskolin, we observed a significant delay in recovery compared to *grk1b−/−* treated with vehicle (F_(1, 224)_ = 17.82; *p* <0.0001) (Fig. 4*A*). While the extent of the delayed recovery associated with forskolin treatment appears smaller in *grk1b−/−* larvae compared to WT (Fig. 3*B*), it should be noted that deletion of either Grk1b or Grk7a resulted in a significant delay in recovery of the cone photoresponse absent any pharmaceutical intervention (29). However, *grk7a−/−* larvae treated with forskolin displayed no significant change in cone recovery compared to vehicle-treated *grk7a−/−* larvae (F_(1, 340)_ = 2.363; *p* = 0.1252) (Fig. 4*B*). These data suggest that the delay in cone recovery brought about by increased levels of cAMP is mediated by Grk7a rather than Grk1b in zebrafish larvae.

### cAMP-dependent phosphorylation of Grk1 is undetectable in vertebrate cone photoreceptors

**Figure 5:**
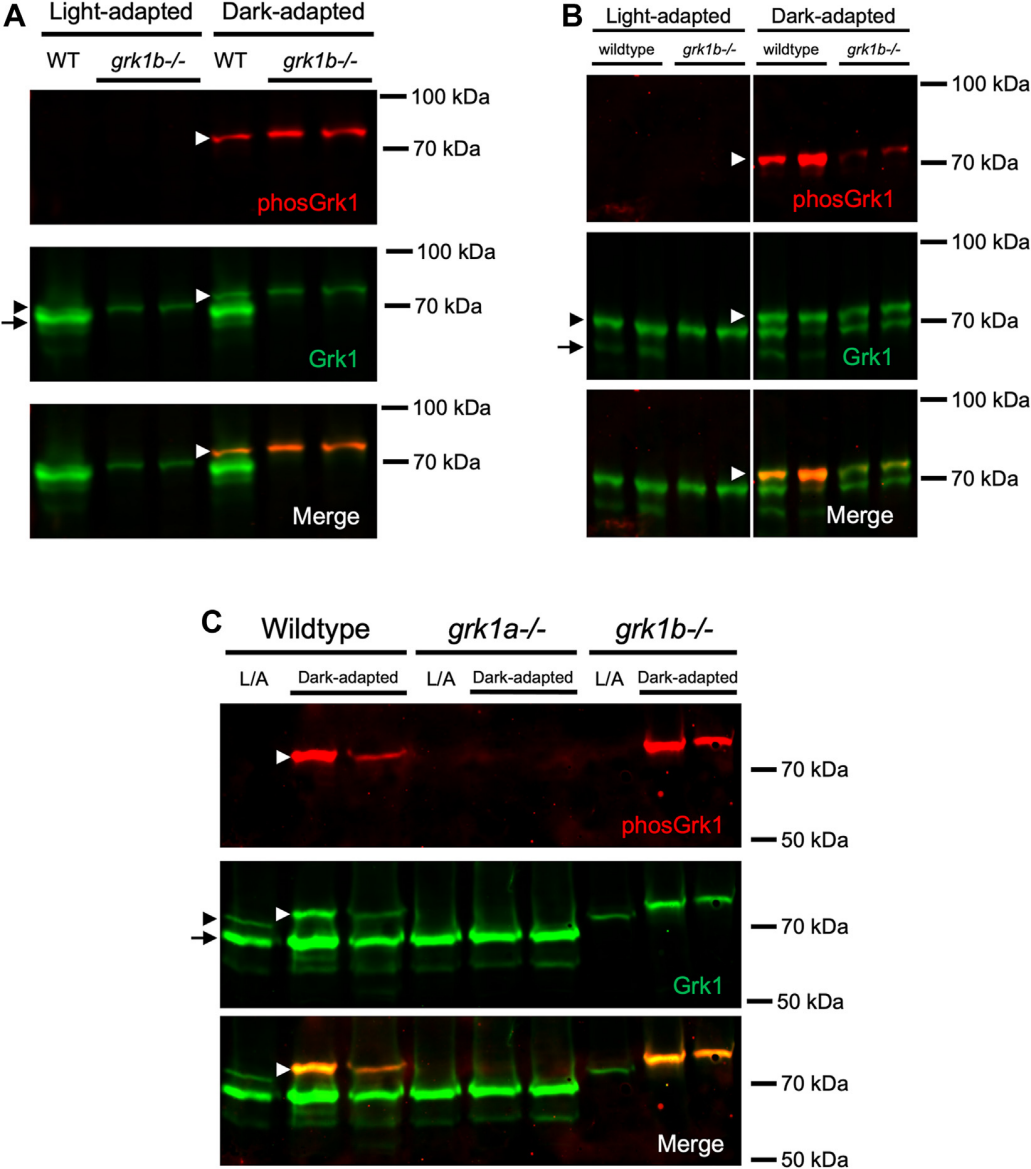
**Grk1 phosphorylation in rod *grk1a* and cone *grk1b* KO zebrafish.** Immunoblots were probed with antibodies against total Grk1 (*green*) and phosphorylated Grk1 (*red*), followed by incubation with secondary antibodies. Comparison of Grk1 phosphorylation in (*A*) light- or dark-adapted WT and *grk1b−/−* larvae at 5 dpf, (*B*) light- or dark-adapted WT and *grk1b−/−* adults, and (*C*) light- or dark-adapted WT, *grk1b−/−*, and *grk1a−/−* larvae at 5 dpf. *Black arrowhead: Grk1a; Black arrow: Grk1b; White arrowhead*, *phosphorylatedGrk1a*. Panel *B* was spliced for clarity due to noncontiguous loading on the same gel.

Our observation that Grk7a plays a greater role than Grk1b in modulating cone opsin lifetime *in vivo* in response to cAMP led us to re-examine the nature of Grk1 phosphorylation in rods and cones. In order to determine whether cone-specific Grk1 is phosphorylated in response to changes in cAMP *in vivo*, the immunoblot analysis was modified to distinguish the Grk1 paralogs. Using the previously characterized *grk1b−/−* fish along with a longer SDS-PAGE running period (Fig. 5, *A* and *B*) or a lower percentage acrylamide gel (Fig. 5*C*) allows for specific identification of immunoblot bands corresponding to each zebrafish Grk1 paralog (Fig. 5). Rod-expressed Grk1a (black arrowheads) migrates more slowly than cone-expressed Grk1b (black arrows), consistent with their predicted molecular weights (Fig. 5*A*). The lower band corresponding to Grk1b is undetectable in *grk1b−/−* fish and the ratio of Grk1a to Grk1b expression is higher in adult zebrafish (Fig. 5*B*) than in larval zebrafish (Fig. 5*A*), most likely because the rod to cone ratio is higher in zebrafish adult retinas (35, 36).

**Figure 6:**
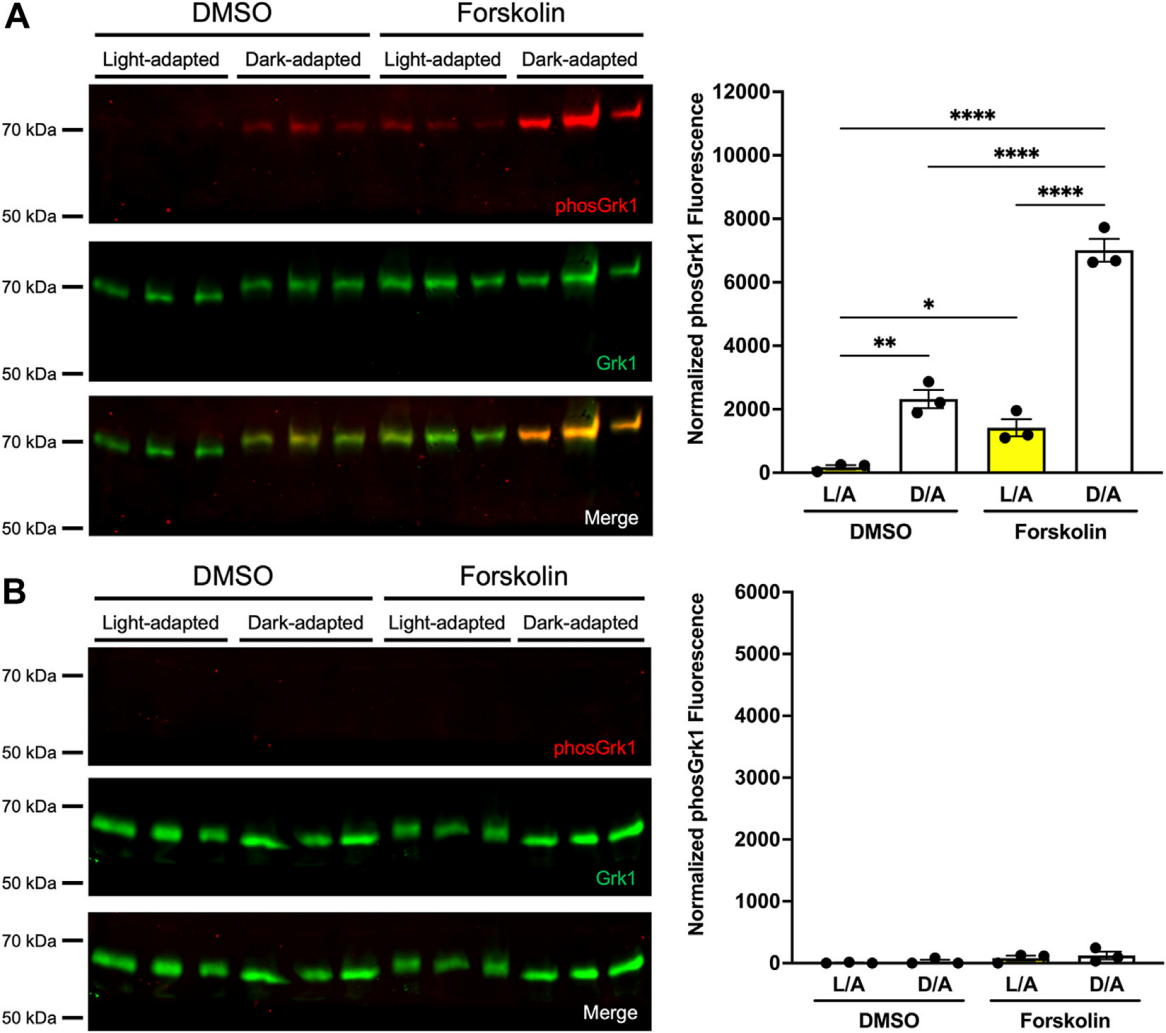
**Effect of light exposure and forskolin incubation on phosphorylation of retinal GRKs in *grk1* KO zebrafish larvae at 5 dpf.***A*, *grk1b−/−* or (*B*) *grk1a−/−* larvae were light or dark adapted, followed by incubation with forskolin or vehicle (dimethyl sulfoxide [DMSO]) for 30 min. Larvae were euthanized and intact eyes were harvested for immunoblot analysis. Immunoblots were probed with antibodies against total Grk1 (*green*) and phosphorylated Grk1 (*red*) followed by incubation with secondary antibodies. The levels of phosphorylated Grk1 were normalized against total Grk1 and quantified. Statistical comparison of multiple groups was performed using a two-way ANOVA followed by a Tukey post hoc test. Error bars represent SEM (n = 3). *p* ≤ 0.05 (∗), *p* ≤ 0.01 (∗∗), *p* ≤ 0.001 (∗∗∗), *p* ≤ 0.0001 (∗∗∗∗).

Since the antibody against phosphorylated GRK1 does not discriminate between the Grk1a and Grk1b paralogs and phosphorylated Grk1 (white arrowheads) is detectable in dark-adapted *grk1b−/−* larvae (Fig. 5*A*) and adults (Fig. 5*B*), it was unclear if both Grk1 paralogs are phosphorylated in a cAMP-dependent manner or just rod-expressed Grk1a. To determine if the phosphorylated Grk1 band detected in fish retinal homogenates is Grk1a, a *grk1a−/−* zebrafish (17bpdel95aa) was generated using CRISPR as described in Experimental procedures. Immunoblot analysis demonstrates that phosphorylated Grk1 is undetectable in dark-adapted *grk1a−/−* fish compared to WT and *grk1b−/−* zebrafish larvae (Fig. 5*C*). When *grk1b−/−* and *grk1a−/−* larvae were treated with forskolin, *grk1b−/−* larvae exhibited a significant increase in Grk1 phosphorylation under both light-adapted and dark-adapted conditions (Fig. 6*A*), whereas no significant Grk1 phosphorylation is detected in forskolin-treated *grk1a−/−* larvae (Fig. 6*B*). Together, these data suggest that only rod-expressed Grk1a undergoes cAMP-dependent phosphorylation in zebrafish.

**Figure 7:**
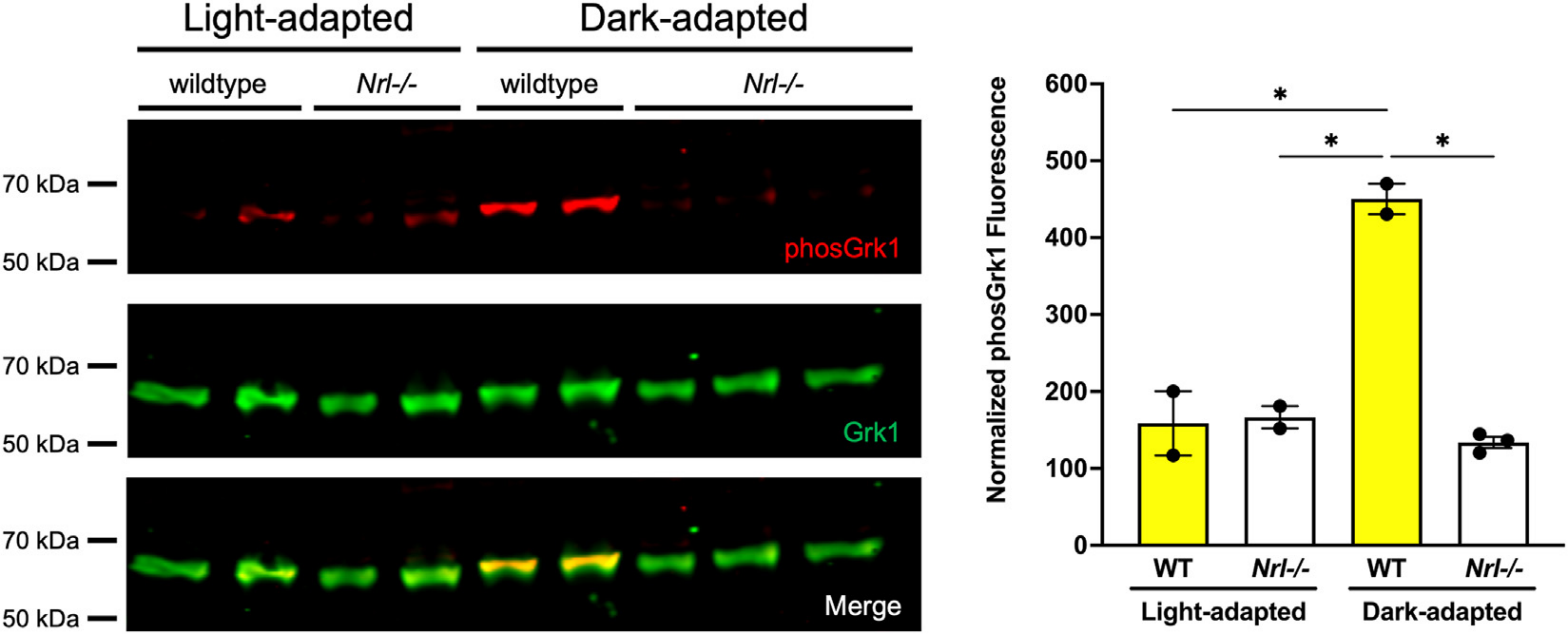
**Grk1 phosphorylation in *Nrl−/−* mice.** Adult mice were light or dark adapted, euthanized, and retinas harvested. Immunoblots were probed with antibodies against total Grk1 (*green*) and phosphorylated Grk1 (*red*), followed by incubation with secondary antibodies. The levels of phosphorylated Grk1 were normalized against total Grk1 and quantified. Statistical comparison of multiple groups was performed using a two-way ANOVA followed by a Tukey post hoc test. Error bars represent SEM, (n=2–3). *p* ≤ 0.05 (∗).

These observations are consistent with our previous report showing that mice with a mutation in Grk1 that converts the phosphorylation site, Ser21, to alanine have delayed dark adaptation (30) in rods but not cones and led us to examine the phosphorylation of Grk1 in the ‘all-cone’ retina of *Nrl−/−* mice. Mice deficient for the Nrl transcription factor possess retinas lacking rods and instead consist mostly of modified S-cones (37). When immunoblots of retinal homogenates of light- or dark-adapted WT and *Nrl−/−* mice were compared, Grk1 was not phosphorylated in dark-adapted *Nrl−/−* mouse retinas compared to WT retinas (Fig. 7). These data suggest that vertebrate GRK1 is consistently phosphorylated only in rods and not in cones.

### Phosphorylation of Grk7 is mediated by PKA *in vivo* in zebrafish cones

**Figure 8:**
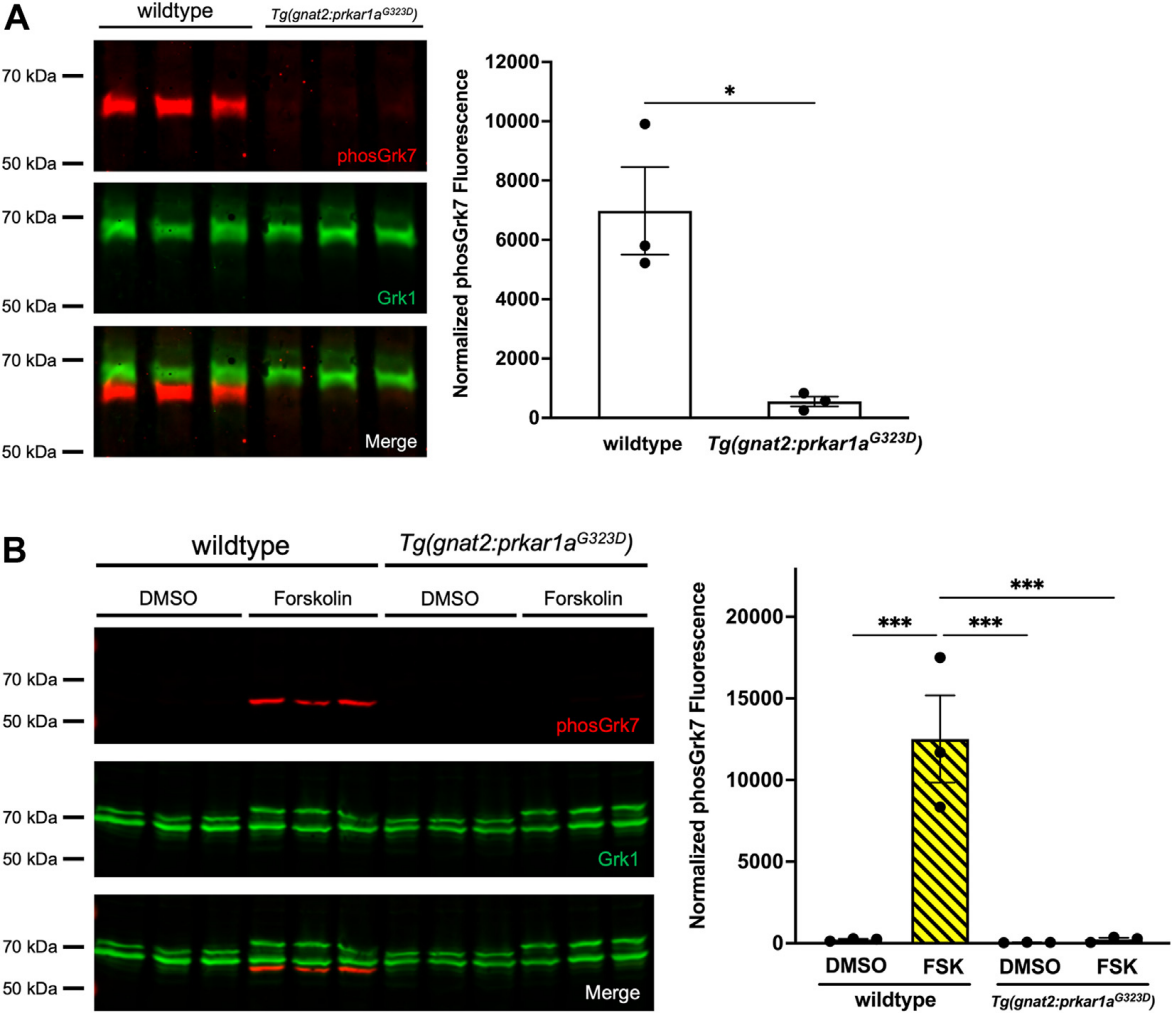
**Dark-adapted Grk phosphorylation in 5 dpf transgenic *Tg(gnat2:prkar1a***^***G323D***^***)* zebrafish larvae expressing PKA dominant negative RI⍺B in cones.** Larvae were (*A*) dark adapted for 15 min or (*B*) light adapted, followed by incubation with forskolin or vehicle (DMSO, dimethyl sulfoxide) for 30 min, then euthanized and intact eyes were harvested for immunoblot analysis. Immunoblots were probed with antibodies against total Grk1 (*green*) and phosphorylated Grk7 (*red*), followed by incubation with secondary antibodies. The levels of detectable phosphorylated Grk7 were normalized against total Grk1 and quantified. Statistical comparison of multiple groups was performed using a Student's *t* test. Error bars represent SEM (n = 3). *p* ≤ 0.05 (∗).

While both Grk1 and Grk7 readily undergo phosphorylation by PKA⍺ *in vitro*, the involvement of PKA in the phosphorylation of retinal GRKs *in vivo* is still speculative. Although PKA is a major downstream effector of cAMP, this cyclic nucleotide can activate signaling pathways through other effectors, such as Epac and cyclic nucleotide-gated ion channels (38, 39). To determine the involvement of endogenous PKA in the phosphorylation of Grk7 in cones, transgenic *Tg(gnat2:prkar1a*^*G323D*^*)* zebrafish expressing a dominant negative RI⍺ regulatory subunit (RI⍺B) that inhibits PKA activation and is driven by the cone transducin promotor (T⍺CP) were generated *via* tol2 transgenesis (40). After light adaption for 2 h, these larvae were dark adapted for 15 min and eyes were enucleated, homogenized, and subjected to immunoblot analysis. After 15 min of dark adaptation, levels of phosphorylated Grk7 were significantly decreased in fish expressing RI⍺B in cone photoreceptors compared to WT fish (Fig. 8*A*). To examine if the expression of RI⍺B in zebrafish cones can block the induction of Grk7a phosphorylation by forskolin under light-adapted conditions, WT and *Tg(gnat2:prkar1a*^*G323D*^*)* larvae were incubated in vehicle or forskolin for 30 min under light-adapted conditions. Compared to WT larvae, *Tg(gnat2:prkar1a*^*G323D*^*)* larvae treated with forskolin exhibited significantly lower levels of Grk7 phosphorylation (Fig. 8*B*). These data suggest that PKA is a critical effector in the phosphorylation of Grk7 in response to increased levels of cAMP in cones.

## Discussion

The present report demonstrates the *in vivo* phosphorylation of the photoreceptor GRKs in vertebrates, as well as the roles they may play in modulating cone phototransduction in response to dynamic intracellular cAMP levels. We chose zebrafish as our principal model, not only because they are an established model for the study of cone photoreceptor kinetics but also because they have a similar distribution profile of photoreceptor Grks as humans. In human retinas, Grk1 is expressed in both rods and cones while Grk7 expression is limited to cones (23). Zebrafish, as a result of a genome duplication event in teleost evolution, express one Grk1 ortholog (Grk1a) in rods and another (Grk1b) in cones and a GRK7 ortholog (Grk7a) in cones (41, 42, 43). The phosphorylation of photoreceptor Grks *in vivo* is likely associated with the elevated levels of intracellular cAMP levels found in dark-adapted photoreceptors, of which PKA is a major downstream effector (19, 20). Additionally, we previously reported that Grk1 phosphorylation is absent in dark-adapted mice when adenylyl cyclase (*Adcy1*) expression is deleted and that *ex vivo* treatment of frog retinas with forskolin is associated with an increase in Grk7 phosphorylation independent of light conditions (17, 18). Using an antibody against phosphorylated Grk7 and an antibody specific for phosphorylated zebrafish Grk1a and Grk1b, we demonstrate that levels of phosphorylated Grk1 and Grk7 are elevated in retinas of dark-adapted zebrafish similar to other species studied.

Frog rod photoreceptors treated with forskolin displayed elevated levels of cAMP in the outer segments, while suction pipette recordings showed increased sensitivity of the rod photoresponse in a manner suggesting that multiple targets are affected that could impact phototransduction (31, 44). This included an increase in PDE activity and a reduction in guanylate cyclase activity, as well as an increase in the Ca^2+^ exchanger current, leading to an increase in intracellular Ca^2+^. Prior studies of increased intracellular Ca^2+^ in rods illustrated its ability to impact light adaptation through several targets such as increased affinity of recoverin for Grk1, decreased guanylyl cyclase activity *via* GCAPs, or a decreased affinity of the cGMP-gated cation channel in outer segments for cGMP *via* inhibition by Ca^2+^-calmodulin (45, 46, 47, 48, 49, 50, 51). Interestingly, a similar study of isolated carp cones treated with forskolin showed that while the rising and descending phases of the light response were slowed, the flash sensitivity of carp cones was not affected in the same manner as rods (52). Using ERG analysis of intact larval zebrafish, we show that forskolin does not affect the normalized sensitivity of the cone photoresponse. However, forskolin is associated with a significant delay in recovery of the cone photoresponse to successive stimuli. Although increased intracellular cAMP likely has several downstream targets that impact the photoresponse, both our *in vitro* and *in vivo* observations of Grk phosphorylation led us to examine the effect of forskolin in zebrafish cone Grk KO lines lacking either Grk1b or Grk7a. We found that while forskolin led to a decrease in the cone photoresponse recovery in *grk1b−/−* larvae, there was no forskolin-associated decrease in cone recovery in *grk7a−/−*. This suggests that modulation of the cone photoresponse by elevated intracellular cAMP is mediated, at least in part, by Grk7a but not Grk1b.

Based on our previous observations that PKA⍺ can phosphorylate GRKs *in vitro* and GRK phosphorylation *in vivo* coincides with elevated intracellular cAMP levels, it is reasonable to suggest that the retinal GRKs are phosphorylated by cAMP-activated PKA *in vivo*. The complete suppression of dark-adapted Grk7a phosphorylation in a zebrafish line expressing a dominant negative inhibitor of PKA in cones is the first proof that PKA plays such a role *in vivo*. While this demonstrates a role for PKA in the phosphorylation of GRK7 *in vivo*, it does not rule out that GRK7 could be phosphorylated by a serine/threonine kinase activated downstream of PKA as has been shown for other proteins (53, 54, 55). Interestingly, while PKA⍺ phosphorylated both GRK1 and GRK7 *in vitro*, our observations of Grk1 phosphorylation in retinas of *grk1a−/−* and *grk1b−/−* zebrafish as well as in the ‘all cone’ retina of the *Nrl−/−* mouse strongly suggest that Grk1 in vertebrate cones is not phosphorylated in a cAMP-dependent manner. This observation agrees with both our previous report of GRK1-S21A mice displaying a significant delay in dark adaptation in rods but not in cones (30), as well the results of this report showing that Grk7a but not Grk1b modulates recovery of the cone photoresponse in forskolin-treated zebrafish larvae.

The involvement of one or more kinases downstream from PKA with different specificities for each GRK paralog and/or a different expression profile in rods and cones could explain the differential phosphorylation of Grk1 between rod and cone photoreceptors, as well as that of Grk1 and Grk7 in cones. Alternatively, if PKA directly phosphorylates the photoreceptor GRKs, differential phosphorylation could be the result of PKA having different affinities for each GRK paralog. A study using a fluorescent biosensor for PKA in mouse retinas demonstrated that PKA was activated less efficiently in cones than in rods following the extinguishing of a short light stimulus (56), which might explain the differences in Grk1 phosphorylation between rods and cones. Another explanation for the lack of detectable cAMP-dependent Grk1 phosphorylation in cones could be a phosphatase that is likely cone-specific (to account for our observations in *Nrl−/−* mice) and which acts preferentially toward Grk1 rather than Grk7 (to account for our observations in zebrafish). Currently, the identity of any phosphatase that targets retinal GRKs is unknown. Previous work by our group showed that inactivation of the autophosphorylation sites important for kinase activity (K219 in GRK1 and K220 in GRK7) did not negatively affect phosphorylation at Ser21 by PKA *in vitro* or in cultured cells incubated with forskolin, suggesting that there are no obvious inhibitory phosphorylation sites on the retinal GRKs (16).

Grk1 phosphorylation could also be sterically hindered in cones but not rods. Aside from its activity towards light-activated opsin, the most widely studied interaction with Grk1 has been with recoverin. When intracellular Ca^2+^ levels are high, extrusion of a myristoyl switch from recoverin exposes a hydrophobic pocket that binds to the first 25 amino acids of Grk1 and blocks the interaction of the kinase with light-activated opsin (13, 14, 15). Considering that the same isoforms of recoverin and Grk1 are expressed in mammalian rods and cones, any potential steric inhibition of Grk1 phosphorylation by recoverin is likely to be associated with cone-specific differences in either recoverin expression levels or Ca^2+^ levels in response to light. In zebrafish photoreceptors, where multiple paralogs of recoverin are differentially expressed, there is greater likelihood that paralog-specific differences in recoverin–Grk1 interaction could account for Grk1 phosphorylation in rods but not in cones (57, 58). Recent analysis using surface plasmon resonance has shown that zebrafish rcv1a, an ortholog of mouse recoverin that is expressed in rods and UV cones, appears to bind equally well to Grk1b in the presence or absence of Ca^2+^ (59). Studies of carp and frog photoreceptors showed that visinin, another neuronal calcium sensor in the same family as recoverin, was expressed at a much higher level in cones compared to recoverin in rods and showed greater inhibition of Grk7 activity compared to Grk1 (60). Despite these differences, the same study reported that the level of inhibition of retinal Grks by visinin was indistinguishable from inhibition by recoverin (60). Additionally, the lack of cAMP-dependent phosphorylation of GRK1 in cones is also observed in mice, which do not express visinin. It is also possible that an unidentified GRK1-interacting protein expressed in vertebrate cones could prevent phosphorylation in response to elevated intracellular cAMP.

While the effects of GRK phosphorylation on the recovery rate of the cone photoresponse appear relatively modest when compared to rods, this underlies the complexity and necessity of the role played by the reversible phosphorylation of photoactivated cone opsins by GRKs for timely adaptation (61, 62). Deletion of GRK1 in mouse cones leads to a profound delay in recovery of the cone photoresponse, while deletion of either Grk1 or Grk7 in zebrafish leads to a similarly significant decrease in cone recovery (29, 63). Interestingly, under dim light, overexpression of GRK1 in mouse cones was associated with a delayed cone response inactivation, whereas reduced GRK1 expression coincided with faster cone response termination. Under brighter stimulation, however, the expression level of GRK1 has no effect on cone response kinetics compared to WT (62). With the molar ratio of GRK1 to cone opsin pigment in mouse cones being approximately 1:100, it is likely that under high light intensities the capacity of GRKs to inactivate phototransduction reaches it upper limits and timely inactivation of the cone response is dominated by the relatively fast spontaneous decay of photoactivated cone opsin (64, 65, 66, 67, 68). The observed lack of GRK1 phosphorylation in cones, by removing a recoverin-independent mechanism of regulation, may in part explain the relatively faster initial rate of opsin phosphorylation observed in carp cones compared to rods (12, 69). It might also explain the slightly slowed dim light cone deactivation kinetics but normal bright light cone responses observed in Oguchi patients with inactivating GRK1 mutations, despite the presence of GRK7 in human cones (28, 70). Future studies are needed to elaborate the role of GRK7 phosphorylation in termination of the cone photoresponse.

In summary, our results show that intracellular cAMP levels modulate recovery of the cone photoresponse *via* PKA-mediated phosphorylation of Grk7 rather than Grk1 in zebrafish, a species with a retinal GRK expression profile similar to humans, and that cAMP-dependent phosphorylation of Grk1 is absent in cones in both zebrafish and mouse. These observations not only corroborate previous reports detailing the role of cAMP-dependent phosphorylation of retina GRKs *in vivo* and *in vitro* but will also help us to better understand the mechanisms of cone adaptation and recovery in relation to human photoreceptor biology.

## Experimental procedures

### Animal studies

All studies utilizing mice and zebrafish have been approved by the Institutional Animal Care and Use Committee of the University of North Carolina at Chapel Hill, which is a PHS Approved Animal Welfare Assurance institution.

### Materials

Mouse monoclonal antibodies (mAbs) against GRK1 for detection in zebrafish tissue (G8, #MA1-720) and for detection in mouse tissue (D11, #MA1-721), as well as secondary antibodies for immunoblot analysis (Alexa Fluor 680 goat anti-rabbit IgG, #A-21076) and immunocytochemistry (Alexa Fluor 488 goat antimouse IgG, #A-11001, and Alexa Fluor 594 goat anti-rabbit IgG, #A-11012) were purchased from ThermoFisher Scientific. IRDye800 goat antimouse IgG (#926-32210) secondary antibody and Intercept (PBS) blocking buffer (#927-70001) were purchased from Licor Biosciences. A mouse mAb against FLAG-tag (#F1804) was purchased from MilliporeSigma. A rabbit polyclonal against phosphorylated GRK7 was generated by Bethyl Laboratories Inc (17). Rabbit polyclonal antibodies against zebrafish Grk7 (29) and phosphorylated mouse Grk1 (18) were generated by 21st Century Biochemicals. A novel rabbit polyclonal antibody against phosphorylated zebrafish Grk1 was also generated by 21st Century Biochemicals using the peptide ISARG[pS]FDGTAN corresponding to amino acids 16 to 27 of zebrafish Grk1a. Mix-n-Stain CF Dye Antibody Labeling Kits (CF-680, #92282, and CF-770, #92285) were purchased from Biotium.

### Generation of *grk1a−/−* zebrafish line

CRISPR single-guide RNAs (sgRNAs) targeting exon 1 of zebrafish *grk1a* were designed using CHOPCHOP (https://chopchop.cbu.uib.no/) and generated based on the methods in Hwang *et al*. (71). Sense and antisense oligonucleotides corresponding to each target sequence were ordered from Integrated DNA Technologies and 1 nmol of each was annealed for 4 min at 95 °C in annealing buffer with a final concentration of 6 mM Tris (pH 7.5), 30 mM NaCl, and 600 μM EDTA. Once cooled, annealed oligos were phosphorylated *in vitro* with T4 polynucleotide kinase (#M0201S) from New England Biolabs and ligated into pDR274 vector (#42250) from Addgene, which was previously linearized with BsaI (#R0535, New England Biolabs). Following *in vitro* dephosphorylation using calf intestinal alkaline phosphatase (#M0290, New England Biolabs), the construct was purified with a Qiaquick PCR purification Kit (#28104) from Qiagen. Following transformation into Dh5⍺ cells, colony selection, and screening by Sanger sequencing (Genewiz), plasmid clones containing the correct insert were linearized with DraI (#R0129S, New England Biolabs) and sgRNA was synthesized *in vitro* using a MAXIscript T7 Transcription Kit (#AM1312, ThermoFisher Scientific). Following cleanup of the sgRNAs using Micro Bio-Spin 6 columns (#7326221; Bio-Rad) and quantification, 80 pg of sgRNA was coinjected with 200 pg of Cas9 protein (#CP01-50; PNA Bio) into 1-cell zebrafish embryos. Genomic DNA was prepared from uninjected and injected larvae at 5 dpf and the induction of indels was measured by high resolution melt analysis (HRMA). The sgRNA with the highest efficiency was 5′-GGACGTAGAGGAATACGACACGG-3’. Fish injected with this sgRNA were raised to sexual maturity (F0) and outcrossed to WT fish to create the F1 generation. F1 heterozygote larvae at 5 dpf were analyzed by HRMA to find an F0 parent with germline transmission and by Sanger sequencing to determine the sequence of the indel mutations. An F0 founder was identified that contained a 17 bp deletion in exon 1 of *grk1a* giving rise to an early termination codon at Tyr96 (pTyr96∗). Additional F1 progeny from this fish were raised to sexual maturity and fin-clipped to find individuals carrying the pTyr96∗ allele. Once identified, heterozygous pTyr96∗ F1 fish were in-crossed to produce F2 offspring. These F2 fish were raised to adulthood and fin-clipped for HRMA and Sanger sequencing to identify homozygous pTyr96∗ fish, which were used to continue the line, hereafter designated *grk1a−/−*.

### Generation of a PKA dominant negative *Tg(gnat2:prkar1a*^*G323D*^*)* zebrafish line

The dominant negative PKA RI⍺B mutant was originally generated by a G324D mutation in the *Prkar1a* gene encoding the RI⍺ regulatory subunit of PKA in mice (40, 72). This glycine is conserved in the zebrafish ortholog *prkar1aa*, at position 323. After cloning *prkar1aa* from a zebrafish retina complementary DNA library and inserting into pGemT-Easy vector (#A1360) from Promega, site-directed mutagenesis was performed as described in Liu & Naismith (73) using primers 5′-ACTACTTCGATGAGATCGCTCTGCTCATGAACCGTCCTCGTGCTG and 5′-AGCGATCTCATCGAAGTAGTCAGACGGTGCGAGTCTTCCAACCTC with Phusion Plus DNA polymerase (#F630S, ThermoFisher). Following confirmation of the RI⍺B G323D mutation by Sanger sequencing, the zebrafish RI⍺B fragment was cloned into the pME-MCS (#237) middle entry vector of the tol2kit (74). Simultaneously, a 3.2-kb promoter fragment (T⍺CP) from cone transducin alpha (*gnat2*) (75) was cloned into p5E-MCS 5′ entry vector (#228). Gateway cloning was performed using LR Clonase II Plus enzyme mix (#12538200, ThermoFisher) with these two entry clones plus the p3E-polyA (#302) 3′ entry vector and the pDestTol2CG2 (#395) destination vector. Successful recombination of the T⍺CP-RI⍺B-polyA-pDestTol2CG2 plasmid was confirmed by Sanger sequencing of transformed clones. Capped Tol2 transposase mRNA was synthesized *in vitro* using the mMessage mMachine SP6 Transcription Kit (#AM1340, ThermoFisher) with NotI-linearized pCS2FA-transposase (#396) plasmid as a template. The T⍺CP-RI⍺B-polyA-pDestTol2CG2 plasmid (30 pg) and Tol2 transposase mRNA (25 pg) were coinjected into zebrafish embryos at the 1-cell stage. Live injected embryos were screened using fluorescence microscopy at 4 dpf to identify EGFP mosaic expression in cardiac muscle, as the pDestTol2CG2 destination vector also carries *cmlc2*:EGFP as a transgenesis marker. EGFP-positive larvae were raised to sexual maturity (F0) and outcrossed to WT fish to produce F1 zebrafish, which were similarly screened for heart EGFP expression to identify germline transmitting F0 founders. F1 zebrafish and subsequent generations that were EGFP-positive were used in these studies. Animals from this line are designated *Tg(gnat2:prkar1a*^*G323D*^*)*.

### Immunoblotting

Unless noted, all tissue samples for immunoblot analysis were collected in Hepes-Ringer buffer containing 10 mM Hepes, pH 7.5, 120 mM sodium chloride, 0.5 mM potassium chloride, 0.2 mM calcium chloride, 0.2 mM magnesium chloride, 0.1 mM EDTA, 10 mM glucose, and 1 mM DTT (76). Hot (95 °C) 2× Laemmli buffer (125 mM Tris–HCl pH 6.8, 4% SDS [w/v], 20% glycerol [v/v]), without bromophenol blue or β-mercaptoethanol but containing 100 μM NaF to block protein dephosphorylation, was added to a final concentration of 1.2× (77). Samples were homogenized briefly with a motorized pestle, heated for 5 min at 95 °C, sonicated at low power for 5 s to shear genomic DNA, heated again for 2 min at 95 °C, and the supernatant collected following centrifugation at 10,000*g* for 7 min. Protein concentration was determined using a DC Protein Assay (#5000112) from Bio-Rad. Following addition of bromphenol blue and β-mercaptoethanol (final concentrations 0.01% [w/v] and 5% [v/v], respectively), homogenates were analyzed by either 8% or 10% SDS-PAGE, followed by immunoblot analysis. Spectra Multicolor Broad Range Protein Ladder (#26634, ThermoFisher) was loaded in a lane of all gels/blots as a molecular weight marker. Nitrocellulose membranes were blocked in Intercept (PBS) Blocking Buffer (#927-70001, Licor Biosciences) and incubated overnight at 4 °C in Intercept buffer with 0.1% Tween 20 (v/v) and primary antibodies. Blots were washed four times for 5 min each in 1× PBS containing 0.1% Tween 20, then incubated in Intercept buffer containing 0.1% Tween 20 and secondary antibodies for 1 h at room temperature. Following four additional washes in 1× PBS containing 0.1% Tween 20 for 5 min each and one wash in 1× PBS for 5 min, blots were analyzed using the Odyssey Infrared Imaging System (Licor Biosciences). All primary antibodies were used at a dilution of 1:10,000 except for anti-phosGrk7 (1:2000), and all secondary antibodies were used at a dilution of 1:15,000. For experiments requiring the use of two antibodies derived from the same host species, primary antibodies were directly conjugated to fluorophores using Mix-n-Stain CF Dye Antibody Labeling Kits (Biotium) according to the manufacturer’s instructions.

To determine levels of phosphorylation of retinal GRKs, tissue preparations varied by species and age. For zebrafish adults, both retinas were harvested and placed in 100 μl of Hepes-Ringer buffer to which 150 μl of hot (95 °C) 2× Laemmli buffer was added, then processed as described above. Following protein quantification, 25 μg of total protein per adult zebrafish retina sample was run in each lane. For 5 dpf zebrafish larvae, whole intact eyes from 20 larvae were harvested and placed in 16 μl of ice-cold Hepes-Ringer buffer to which 24 μl of hot (95 °C) 2× Laemmli buffer was added, then processed as described above. An entire zebrafish larval eye sample (40 eyes) was loaded in each lane.

To determine the immunoreactivity of the novel anti-phosphoGrk1 antibody, recombinant FLAG-tagged zebrafish Grk1a and Grk1b were each inserted into the pFLAG-CMV2 vector (#E7033, Millipore-Sigma). HEK-293 cells cultured in DME/F-12 with 10% fetal bovine serum were transfected with either of these constructs using FuGENE 6 transfection reagent (#E2691) from Promega. Approximately 72 h after transfection, cells were harvested, frozen once, then homogenized in Tris-buffered saline containing 0.5% n-dodecylmaltoside and cOmplete Protease Inhibitor Cocktail (#11836170001, Millipore-Sigma), followed by centrifugation at 40,000*g* for 30 min at 4 °C. FLAG-tagged zebrafish Grk1a or Grk1b was isolated using anti-FLAG M2 affinity gel (#A2220) from Millipore-Sigma as described previously (16). *In vitro* phosphorylation or dephosphorylation reactions were carried out by incubating FLAG-purified Grk1a or Grk1b with PKA⍺ catalytic subunit (#P6000S) or λ phosphatase (P0753S) from New England Biolabs. Reactions were stopped by the addition of 2× Laemmli buffer to a final concentration of 1.2×, then subjected to immunoblot analysis.

### Electroretinogram (ERG) analysis

ERG analysis was performed as described previously (29, 78, 79). Zebrafish larvae were dark adapted overnight prior to analysis. All larvae were incubated for 5 min under indirect dim white light (<1 lux) in zebrafish system water containing 500 μM 2-amino-4-phosphonobutyric acid (APB) (DL-AP4, #0101) from Bio-Techne/Tocris, an mGluR6 agonist that blocks signaling to ON bipolar cells and allows measurement of the isolated cone mass receptor potential. Larvae were anesthetized with 0.02% Tricaine, positioned under low light (275 lux for ≤1 min) on a piece of filter paper, and covered with 3% methylcellulose posterior to the eye. Specimens on filter paper were then transferred to a damp sponge saturated with oxygenated Goldfish Ringer's buffer (80) on a modified stage underneath a Colordome light stimulator (Diagnosys LLC). The entire ERG apparatus was located inside a black curtained, copper mesh lined Faraday cage. The microelectrode, a chlorided silver wire in a glass capillary with a 10 μm tip opening filled with Goldfish Ringer's buffer, was positioned onto the central cornea. An AgCl pellet underneath the sponge served as a reference electrode. Once positioned, larvae were allowed to dark-adapt for 5 min while monitored with a combination of indirect infrared lighting, an infrared camera and a red-filtered video monitor placed outside the cage. Recordings were obtained using an Espion E2 system (Diagnosys LLC). The sensitivity of the cone mass receptor potential was determined using a single flash paradigm consisting of 20-ms flashes of white LED light with intensities of 1, 5, 10, 20, 50, 100, 500, 1000, 2000, and 5000 cd/m^2^. Recovery to successive stimuli was determined using a dual flash paradigm that utilized two 20-ms flashes of white LED light with an intensity of 1000 cd/m^2^ separated by variable ISIs of darkness.

### Light/dark adaptation

Animals indicated as ‘dark-adapted’ were dark adapted overnight for a minimum of 16 h. All dissections and sample preparations were performed under oblique dim red or infrared light until after the sample was homogenized and heated as described. Animals indicated as ‘light-adapted’ were dark adapted overnight, then placed under white light of 1200 lux for at least 2 h. In the experiment involving the *Tg(gnat2:prkar1a*^*G323D*^*)* transgenic larvae, animals were dark adapted overnight, light adapted under white light (1200 lux) for at least 2 h, then dark adapted for 15 min prior to whole eye collection under oblique dim red or infrared light. The samples were then processed for immunoblot analysis as described.

### Forskolin treatments

For immunoblot analysis, larvae at 5 dpf were incubated in system water for 30 min with either forskolin (50 μM) or vehicle dimethyl sulfoxide (DMSO) (0.5%, v/v). For ERG analysis, larvae were incubated in forskolin or vehicle for 25 min, followed by a 5 min coincubation with APB (500 μM).

### Statistical analyses

For single flash ERGs, normalized peak amplitude responses were fit using the Naka Rushton function with GraphPad Prism (GraphPad Software Inc) (81). For dual flash ERGs, we fit linear mixed models of covariance to compare response recovery between drug treatments across repeated ISI levels. Models included random fish effects to account for within-fish correlations. These analyses were performed using JMP (SAS). For immunoblots, statistical comparison of multiple groups was performed using a two-way ANOVA followed by a Tukey post hoc test. Comparisons in Figure 8 were performed using a Student's *t* test. For all analyses, a *p*-value of 0.05 was considered significant.

## Data availability

All data are contained within the article.

## Supporting information

This article contains supporting information.

## Conflict of interest

The authors declare that they have no conflicts of interest with the contents of this article.
